# Cis-2-Decenoic Acid and Bupivacaine Delivered from Electrospun Chitosan Membranes Increase Cytokine Production in Dermal and Inflammatory Cell Lines

**DOI:** 10.3390/pharmaceutics15102476

**Published:** 2023-10-17

**Authors:** Zoe Harrison, Emily C. Montgomery, Joshua R. Bush, Nidhi Gupta, Joel D. Bumgardner, Tomoko Fujiwara, Daniel L. Baker, Jessica Amber Jennings

**Affiliations:** 1 Department of Biomedical Engineering, University of Memphis, Memphis, TN 38152, USA; zoeharrison0@gmail.com (Z.H.); cclman22@memphis.edu (E.C.M.); josh.bush@memphis.edu (J.R.B.); ngupta1@memphis.edu (N.G.); jbmgrdnr@memphis.edu (J.D.B.); 2 Department of Chemistry, University of Memphis, Memphis, TN 38152, USA; tfjiwara@memphis.edu (T.F.); dlbaker@memphis.edu (D.L.B.)

**Keywords:** electrospun chitosan membrane, wound dressings, bupivacaine, cis-2-decenoic acid, drug release, nanofiber

## Abstract

Wound dressings serve to protect tissue from contamination, alleviate pain, and facilitate wound healing. The biopolymer chitosan is an exemplary choice in wound dressing material as it is biocompatible and has intrinsic antibacterial properties. Infection can be further prevented by loading dressings with cis-2-decenoic acid (C2DA), a non-antibiotic antimicrobial agent, as well as bupivacaine (BUP), a local anesthetic that also has antibacterial capabilities. This study utilized a series of assays to elucidate the responses of dermal cells to decanoic anhydride-modified electrospun chitosan membranes (DA-ESCMs) loaded with C2DA and/or BUP. Cytocompatibility studies determined the toxic loading ranges for C2DA, BUP, and combinations, revealing that higher concentrations (0.3 mg of C2DA and 1.0 mg of BUP) significantly decreased the viability of fibroblasts and keratinocytes. These high concentrations also inhibited collagen production by fibroblasts, with lower loading concentrations promoting collagen deposition. These findings provide insight into preliminary cellular responses to DA-ESCMs and can guide future research on their clinical application as wound dressings.

## 1. Introduction

Biopolymer wound dressings have proven effective in reducing bacterial contamination following surgery or musculoskeletal injury while also enhancing wound healing [1,2]. A functional wound dressing maintains an optimal environment for tissue healing by easily conforming to a patient’s body, providing a barrier to external contamination, and preventing excessive inflammation [3]. Further, it is beneficial for these dressings to be capable of point-of-care loading with antimicrobial or anesthetic agents, making them tailorable to each patient’s unique needs. Thus, to optimize the wound healing response, it is necessary to manufacture dressings from biocompatible materials and load them with agents that are cytocompatible and support regrowth of dermal cells.

During wound healing, dermal cells use sequential signals and responses to properly choreograph tissue repair, which occurs in three major phases: the lag phase, proliferation phase, and remodeling phase [4]. The lag phase begins after injury with the formation of a fibrin and fibronectin blood clot and recruitment of platelets which release chemokines to recruit inflammatory cells, neutrophils, and macrophages, as well as fibroblasts and endothelial cells [5]. This is followed by the proliferation phase, which consists of epithelial keratinocyte cell migration and fibroblast proliferation and concludes with the regeneration phase of collagen deposition [6]. The vitality of keratinocytes in the wound bed is essential as they re-epithelialize damaged tissue and work together with fibroblasts to cover wounds and promote closure. Keratinocytes also release a number of cytokines, such as pro-inflammatory IL-1 and IL-6 and anti-inflammatory IL-10, as well as various chemokines that recruit monocytes and speed up the healing process [7]. In addition to helping keratinocytes cover the wound, fibroblasts also assist in wound healing by breaking down the fibrin clot and synthesizing new extracellular matrix and collagen structures. Thus, keratinocytes and fibroblasts serve as key players that work in union to coordinate the wound healing process.

Chitosan, a polycationic polysaccharide, is a promising material in the manufacture of wound dressings because it is biocompatible, biodegradable, and can be chemically modified to optimize drug loading and release characteristics [8]. Previous work has investigated many chitosan products, from sponges to pastes to electrospun membranes, all of which were capable of point-of-care loading and release of various therapeutics [9,10,11,12,13]. Electrospinning chitosan membranes result in a random distribution of nanoscale fibers with a high surface area and porosity, similar to the structure of a native extracellular matrix [14]. These characteristics also allow for high-volume drug loading as well as continued exchange of fluids and nutrients [15]. Furthermore, the nanostructural properties of the electrospun chitosan membranes (ESCMs) allow cell growth but not complete infiltration within intermembrane pores.

The membranes investigated in this study use a decanoic anhydride acylation treatment for ESCMs (DA-ESCMs), a modification of the procedure developed by Wu et al. to allow for loading of hydrophobic local anesthetic bupivacaine (BUP) and the anti-biofilm fatty acid cis-2-decenoic acid (C2DA) [16]. BUP is a local anesthetic with intrinsic antimicrobial properties that is clinically used in the forms of topical creams, ointments, and sprays [17,18]. Loading membranes with BUP provides local pain management, thus decreasing the need for systemic opioid use. C2DA is a known biofilm dispersal agent that can prevent wound infection and colonization by multiple bacterial strains [19]. Previous work has demonstrated the efficacious release of both BUP and C2DA from hexanoic anhydride-treated ESCMs (HA-ESCMs), as well as their prevention of *Staphylococcus aureus* biofilm growth, though high loading levels were cytotoxic to mouse fibroblasts, L929 [13].

This study sought to evaluate human dermal and inflammatory cell responses to BUP, C2DA, and combinations released from DA-ESCMs. DA-ESCMs are preferred over HA-ESCMs because a longer chain length leads to more hydrophobicity and greater interaction with carbon chains, extending the release of hydrophobics [20]. Initially, BUP and C2DA loading concentrations were adjusted to prevent the cytotoxic effects reported in previous studies [13]. DA-ESCMs were loaded with a range of C2DA and BUP concentrations, either individually or in combination, and tested with keratinocytes and fibroblasts to determine cytocompatible therapeutic loading concentrations. Collagen production of fibroblasts in contact with DA-ESCMs was measured to determine the effects of loading concentration on fibroblast function. We hypothesize that compatibility and collagen production will vary based on therapeutic loading, with higher concentrations correlating with higher toxicity.

## 2. Materials and Methods

### 2.1. Materials

Chitosan flakes (86% DDA) were purchased from Primex (Siglufjordur, Iceland). C2DA was synthesized by the authors as previously described [21]. NHDFs, NHEKs, and their respective media and additives were purchased from Lonza (Basel, Switzerland). CellTiter-Glo was purchased from Promega (Madison, WI, USA). Sirius Red Total Collagen Detection Kit was from Chondrex (Woodinville, WA, USA). Bupivacaine and other chemical reagents were purchased from MilliporeSigma (Darmstadt, Germany).

### 2.2. Membrane Fabrication

Membranes were electrospun using a 71% degree of deacetylation, 311.5 kDa chitosan (Primex) at 5.5% (*w*/*v*) in 70% (*v*/*v*) trifluoroacetic acid (TFA)—30% (*v*/*v*) dichloromethane solution at 26 kV, as previously described. Membranes were spun to 15 cm diameters and ~0.7 mm (30 mL of spinning solution) thickness and treated using a 50–50 solution of pyridine and decanoic anhydride. Membranes were punched into 1 cm diameter discs and UV-sterilized prior to contact with cells. Ethanol (200 proof) was used for dissolving therapeutics, and membranes were loaded by pipetting 30 µL of a stock solution onto the surface so that a known mass was added to the membrane: C2DA (0.075, 0.15, 0.3 mg), BUP (0.25, 0.5, or 1.0 mg), or a combination of both treatments (Table 1). After therapeutic concentrations were applied to membranes, the membranes were dried aseptically in a laminar flow hood to allow ethanol evaporation, leaving therapeutics incorporated within the membrane fibers.

### 2.3. Cytocompatibility

Normal adult human dermal fibroblasts (NHDFs, Lonza) were cultured in FBM-2 Basal Medium plus FGM-2 SingleQuots supplements (Lonza), normal adult human epidermal keratinocytes (NHEKs, Lonza) in KBM-Gold Keratinocyte Growth Basal Medium plus SingleQuots Supplements and Growth Factors (Lonza) and RAW 264.7 monocytes (ATCC, Manassas, VA, USA) in DMEM plus 10% FBS. All cells were seeded at 1 × 10^4^ cells/cm^2^ in 24-well plates and cultured for 24 h at 37 °C and 5% CO_2_ before adding the experimental treatment. All media were supplemented with 500 IU/mL penicillin, 500 μg/mL streptomycin, and 2.5 μg/mL amphotericin-B. After overnight incubation, DA-ESCMs were placed in the wells. After 24 and 72 h, wells were imaged microscopically, and cell viability was quantified using the CellTiter-Glo^®^ viability assay (Promega). Results were normalized as a percent viability versus cells grown with unloaded DA-ESCMs. Treatments were accepted as cytocompatible if they met or surpassed the 70% cytocompatibility minimum, as established by ISO 10993-5 [22].

### 2.4. NHDF Collagen Production

Supernatant media from NHDF cytocompatibility assays were used for the determination of collagen production using the Sirius Red Total Collagen Detection Kit (Chondrex, Woodinville, WA, USA). Sirius red is a unique dye which specifically binds to the [Gly-X-Y]n helical structure on fibrillar collagen (type I to V) and does not discriminate between collagen species and types. Briefly, supernatants were treated with a concentrating solution, stained with Sirius red dye, washed with a washing solution, and treated with an extraction buffer, and then optical density was read at 540 nm using a BioTek Synergy plate reader. Cells were centrifuged at 10,000 rpm for 3 min between each assay step. Collagen concentrations were calculated by referencing a standard curve generated by known concentrations of collagen (μg/mL, normalized by corresponding well’s supernatant viability).

### 2.5. Cytokine Production

NHEK and RAW 264.7 supernatants were assayed for IL-10/IL-12/TNF-α/VEGF and IL-10/TNF-α, respectively, using ELISA. ELISA (Peprotech US, Cranbury, NJ, USA) was performed according to the manufacturer’s instructions. Absorbance were read at 450 nm using a BioTek Synergy microplate reader (Agilent Technologies, Santa Clara, CA, USA). Levels of cytokine were expressed as specific units of activity (ng/mL, normalized by corresponding well’s supernatant viability). One group of RAW 264.7 monocytes were treated with 100 ng/mL LPS for 24–72 h to induce stimulation.

### 2.6. Statistical Analysis

Statistically significant differences were tested with an ANOVA followed by Tukey’s multiple comparisons test. Statistical analyses were performed in Prism version 8.4.3 (GraphPad Software, San Diego, CA, USA) at a significance level of 0.05. Data are reported as mean ± standard deviation. Throughout results, * indicates *p* < 0.05, ** indicates *p* < 0.01, *** indicates *p* < 0.001, and **** indicates *p* < 0.0001.

## 3. Results

### 3.1. Normal Adult Human Dermal Fibroblasts (NHDFs)

#### 3.1.1. Cytocompatibility

**Figure 1:**
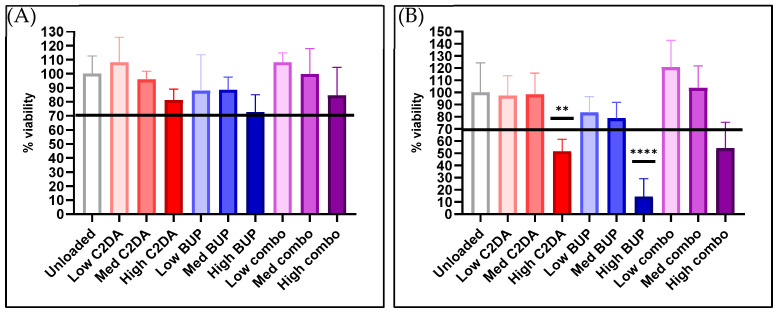
Cytocompatibility of loaded and unloaded DA-ESCMs with normal human dermal fibroblasts. Graphs indicate percent viability of NHDFs in contact with DA-ESCMs for (**A**) 24 h or (**B**) 72 h (*n* = 4). Viability was quantified based on metabolic activity by measuring ATP production. Individual data points are shown as bars representing mean and error bars representing standard deviation. Black line indicates the 70% cytocompatibility minimum established by ISO 10993-5. ** indicates significantly lower viability compared to unloaded control with *p* < 0.01 and **** indicates *p* < 0.0001, detected using one-way ANOVA with Tukey’s post hoc tests.

At 24 h, all membrane groups met or surpassed the cytocompatibility threshold of 70% viability. DA-ESCMs loaded with high concentrations of C2DA and the C2DA/BUP combination were determined to be cytotoxic to NHDFs. Membranes loaded with high concentrations of BUP alone were most cytotoxic to NHDFs at 72 h, with approximately 10% of viable NHDFs remaining compared to unloaded controls. The medium and low BUP and C2DA loadings as well as the low combination loading remained cytocompatible with NHDFs at 72 h (Figure 1).

#### 3.1.2. Collagen Production

**Figure 2:**
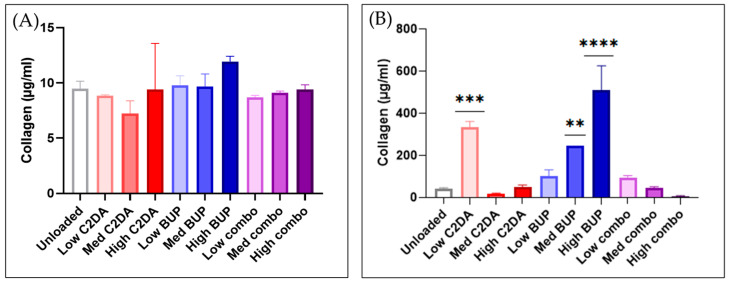
Quantification of collagen production by NHDFs after contact with loaded and unloaded DA-ESCMs as determined using a colorimetric collagen assay. Graphs indicate collagen production (μg/mL) from supernatant media of NHDFs in contact with DA-ESCMs for (**A**) 24 h or (**B**) 72 h (*n* = 4). Individual data points are shown as bars representing mean and error bars representing standard deviation. ** indicates significantly higher collagen production compared to unloaded control, detected using one-way ANOVA with Tukey’s post hoc tests (*p* < 0.01), *** indicates *p* < 0.001, and **** indicates *p* < 0.0001.

Sirius red detection assays indicated minimal collagen production for all groups at 24 h, with all groups producing approximately 10 μg/mL. Fibroblasts treated with unloaded membranes resulted in 50 μg/mL collagen production at 72 h, and similar production levels for fibroblasts treated with a medium concentration of combination membranes and low concentrations of C2DA membranes. Fibroblasts treated with low BUP or low combination concentrations produced approximately 2× the amount of collagen compared to those treated with unloaded membranes. Fibroblasts treated with high concentrations of BUP membranes generated the highest amount of collagen at approximately 500 μg/mL. DA-ESCMs with medium BUP or low C2DA loadings generated approximately 200–300 μg/mL collagen. Finally, fibroblasts treated with medium concentrations of C2DA membranes and high concentrations of combination membranes generated the lowest levels of collagen (Figure 2).

### 3.2. Normal Adult Human Epidermal Keratinocytes (NHEKs)

#### 3.2.1. Cytocompatibility

**Figure 3:**
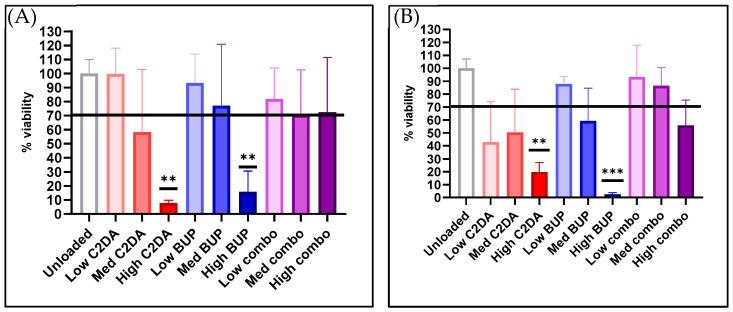
Cytocompatibility of loaded and unloaded DA-ESCMs with normal human epithelial keratinocytes. Graphs indicate percent viability of NHEKs in contact with DA-ESCMs for (**A**) 24 h or (**B**) 72 h (*n* = 4). Viability was quantified based on metabolic activity by measuring ATP production. Individual data points are shown as bars representing mean and error bars representing standard deviation. Black line indicates the 70% cytocompatibility minimum established by ISO 10993-5. ** indicates significantly lower viability compared to unloaded control with *p* < 0.01 and *** indicates *p* < 0.001, detected using one-way ANOVA with Tukey’s post hoc tests.

The results indicate that while the highest concentration of both therapeutics alone was cytotoxic to NHEKs, lower concentrations and simultaneous delivery of both C2DA and BUP were cytocompatible with NHEKs. At 24 h, membranes loaded with high and medium concentrations of C2DA only were cytotoxic to NHEKs. Membranes loaded with low C2DA concentrations were 100% cytocompatible, equivalent to unloaded membranes. Membranes loaded with high concentrations of BUP alone were also cytotoxic at 24 h, but those loaded with medium or low concentrations of BUP were cytocompatible with NHEKs. All groups loaded with both therapeutics combined (high, medium, and low) were cytocompatible with NHEKs at 24 h. At 72 h, similar trends were present; however, cells recovered 10% viability with a high C2DA loading, while high-BUP-concentration membranes remained cytotoxic. At the 72 h timepoint, NHEK responses to each therapeutic loading concentration began to mimic NHDF responses. Simultaneous delivery of both therapeutic molecules showed better levels of cytocompatibility in each group compared to delivering either molecule alone, with medium C2DA and BUP concentrations being almost as cytocompatible as lower concentrations (Figure 3).

#### 3.2.2. Cytokine Production

**Figure 4:**
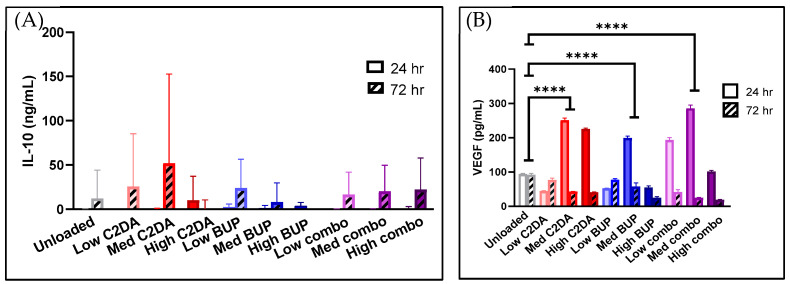
(**A**) IL-10 and (**B**) VEGF production by NHEKs in contact with DA-ESCMs for 24 or 72 h (*n* = 4). Individual data points are shown as bars representing mean and error bars representing standard deviation. Concentrations were normalized based on viability for each sample. **** indicates *p* < 0.0001, detected using one-way ANOVA with Tukey’s post hoc tests.

The NHEK ELISA results indicate that C2DA and BUP, as well as their combinations, triggered a release of 25–50 ng/mL IL-10 from keratinocytes after 24 h, as release occurred at 72 h and not 24 h for most groups (Figure 4). No statistically significant difference was observed between unloaded and loaded DA-ESCMs. ELISAs measuring IL-12/TNF-α did not determine a detectable release for any group. However, after 24 h, the NHEK cells produced significant changes in VEGF production for both medium- and high-concentration groups of C2DA. Production of VEGF appeared to return to baseline by 72 h.

### 3.3. RAW 264.7 Monocytes

#### 3.3.1. Cytocompatibility

**Figure 5:**
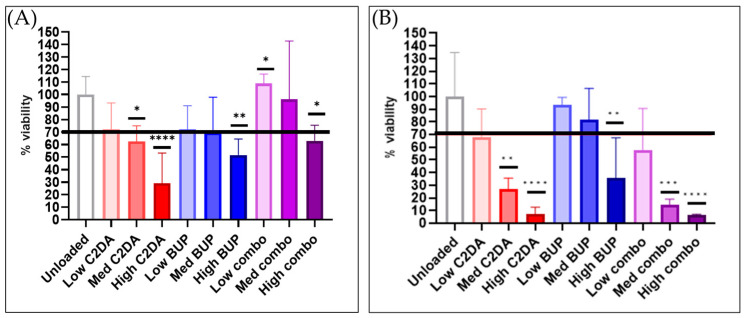
Cytocompatibility of loaded and unloaded DA-ESCMs with murine macrophages. Graphs indicate percent viability of RAW 264.7 cells in contact with DA-ESCMs for (**A**) 24 h or (**B**) 72 h (*n* = 4). Viability was quantified based on metabolic activity by measuring ATP production. Individual data points are shown as bars representing mean and error bars representing standard deviation. Black line indicates the 70% cytocompatibility minimum established by ISO 10993-5. * indicates significantly lower viability compared to unloaded control with *p* < 0.05, ** indicates *p* < 0.01, *** indicates *p* < 0.001, and **** indicates *p* < 0.0001, detected using one-way ANOVA with Tukey’s post hoc tests.

After 24 h, only the high concentrations led to statistically significant decreases in the viability of RAW 264.7 monocytes. However, after 72 h, most groups showed decreases (Figure 5). The combination delivery of high levels of C2DA and BUP did show a recovery in cell viability after 24 h compared to either molecule delivered on its own.

#### 3.3.2. Cytokine Production

**Figure 6:**
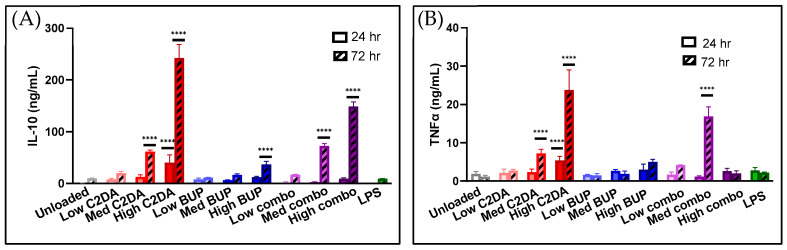
Production of (**A**) IL-10 and (**B**) TNF-α by monocytes in contact with DA-ESCMs for 24 or 72 h (*n* = 4). Individual data points are shown as bars representing mean and error bars representing standard deviation. Concentrations were normalized based on viability for each sample. **** indicates *p* < 0.0001, detected using one-way ANOVA with Tukey’s post hoc tests.

After 72 h, production of both TNF-α and IL-10 increased in the monocyte groups treated with C2DA; however, the levels of IL-10 were much greater than the TNF-α levels. No significant changes were observed after only 24 h (Figure 6). BUP had minimal impact on cytokine production.

## 4. Discussion

Biopolymer wound dressings loaded with antimicrobials and local anesthetics have the dual benefits of preventing contamination and alleviating pain at the wound site. Previous studies of ESCMs have shown their efficacy in drug release and antimicrobial capability against multiple bacterial strains, as well as their effects in preliminary cytocompatibility studies [13]. This study sought to use a combination of C2DA and BUP loaded on DA-ESCMs to determine their combined and individual effects on dermal cell viability and collagen production for wound healing. We confirmed our hypothesis that therapeutic loading concentrations of C2DA and BUP combined greatly affected outcomes synergistically.

Dermal keratinocytes appeared to be more sensitive to the DA-ESCMs in general compared to dermal fibroblasts, a finding that was also noted in a study investigating the responses of NHEKs to polyvinyl alcohol nanofibers [23]. Previous studies showed a higher compatibility of C2DA with NHEKs, though at lower concentrations than were presented in this study [24]. The viability of NHDFs in contact with C2DA-loaded DA-ESCMs was slightly higher than the viability observed in previous studies that directly incubated similar C2DA concentrations with NHDFs and NIH-3T3 fibroblasts cultures [21,25]. This may be due to the sustained delivery of therapeutics from membranes as opposed to the direct inoculation of cells with C2DA and no carrier system. A cytocompatibility study of ESCMs loaded with similar concentrations of C2DA (0.125 and 0.25 mg) also resulted in a high viability of L929 murine fibroblasts after 24 h of contact [26]. The decrease in monocyte viability in response to C2DA is in line with previous studies that showed the short-chain fatty acid n-butyrate causes apoptosis and reduced adhesion of monocytes [27]. The use of leukemic murine monocytes rather than primary human cells is a limitation of this study. The monocytes used in this study were not activated into macrophages, as these wound dressings are intended for immediate use after injury and the goal was to capture preliminary effects within 3 days. Future studies will delve further into the cellular responses of human monocytes and macrophages to C2DA.

ESCMs loaded with higher concentrations of BUP (5.0 and 2.5 mg) in previous studies with murine fibroblasts were far more cytotoxic, solidifying the decision to utilize lower BUP loading concentrations of 0.25, 0.5, and 1.0 mg for human cell testing [13]. The BUP toxicity at 1.0 mg observed with each cell type is consistent with previous studies which cited a low viability of fibroblasts treated with 0.6 mg of BUP [28]. In previous studies, high concentrations of BUP have shown toxicity to keratinocytes, whereas lower concentrations (<0.1 mmol) showed a proliferative effect [29]. Future studies of DA-ESCMs may incorporate lower BUP concentrations to explore this finding further. BUP has been reported to inhibit monocyte viability at 1mmol concentrations (~0.28 mg/mL), which confirms the slightly toxic effect observed with all loading concentrations tested in this study [30]. Further, an in vivo rat model showed that bupivacaine hydrochloride was found to induce macrophage apoptosis [31]. The variability in cytocompatibility results may have been due to inconsistencies in membrane manufacturing; while representative samples from each electrospun membrane were tested for residual TFA, there may have been remnants on select individual punch-outs that altered the cytocompatibility of each sample.

DA-ESCMs loaded with high concentrations of C2DA inhibited collagen production, though the lowest concentration of C2DA stimulated collagen production. Either behavior can benefit burn wound healing depending on the severity of the injury. An over production of collagen results in disorganized scar tissue, yet collagen production is necessary for tensile strength, vascularization, and remodeling of regenerated tissue [32,33]. While collagen production in response to C2DA has not been tested previously, studies of other fatty acids have shown a concentration-dependent relationship between treatment and collagen stimulation, in that high concentrations of fatty acids can inhibit collagen deposition [34,35]. Increased collagen production in response to BUP at all concentrations, as well as increased collagen deposition in fibroblasts treated with medium- and low-concentration combinations of C2DA and BUP compared to those treated with unloaded DA-ESCMs, is consistent with previous studies that have shown that BUP can increase collagen production in vivo to a significantly greater extent than the structurally related local anesthetic ropivacaine [36]. A limitation of this study is that only total collagen produced was measured; type-specific collagen analysis as well as gene expression could provide insight into the mechanisms of fibroblast stimulation or depression by DA-ESCMs. Comparison to other clinically used systems, such as silver-based wound dressings, will be relevant in future studies, particularly for in vivo analysis. In addition, the monocyte cell line used was a mouse cell line, while the rest of the cells used were human cell lines.

Keratinocyte IL-10 secretion appeared dependent on timepoint, as most groups had more detectable levels at the 72 h timepoint; IL-12 was not detected for DA-ESCM-treated cells at either timepoint. While some studies report IL-12 secretion by keratinocytes, other studies report that IL-12 is upregulated in keratinocytes under specific conditions, e.g., following UV-B radiation [37,38,39]. Because data on the production of IL-12 by keratinocytes vary, analyzing the secretion of another pro-inflammatory cytokine, such as IL-1 or IL-6, may benefit future studies. ELISA results indicate high production of both pro-inflammatory TNF-A and anti-inflammatory IL-10. These chemical signals are part of the crosstalk controlling inflammation and mesenchymal stem cell activity [40]. Previous studies have shown the timing of TNF-A and IL-10 release varies, so timepoint for sampling may need to be adjusted in future studies. However, IL-10 production was approximately 10x higher for all groups, indicating that BUP and/or C2DA can stimulate IL-10 release and thus have a dampening effect on TNF-A stimulation. Alternatively, IL-10 has been shown to actually have pro-inflammatory activities in some in vivo works, so designating these cytokines as purely anti- or pro-inflammatory may be shortsighted [41,42]. The present study is limited to mouse RAW cells. Future studies will investigate a complete panel of cytokines for human monocyte and each cell type to further determine the inflammatory responses to DA-ESCMs. Further, LPS at 100 ng/mL did not induce a significant amount of TNF-α compared to the unloaded groups, so a higher dosage will be used in future studies to ensure maximum inflammatory cytokine release from positive control cells. An overall limitation of this study is that cell studies do not fully predict responses in vivo but instead examine cell responses to refine loading for in vivo studies.

## 5. Conclusions

These membranes, which approximate the nanofibrous structure of native ECMs, may prevent further damage and support healing when dressings are applied to cover wounds. Therapeutic concentrations of a biofilm inhibitor and local anesthetic were released to evaluate their ability to protect wounds from biofilm formation, promote non-inflammatory signaling, and support regenerative collagen production profiles. Loading strategies that promote collagen secretion could be beneficial in stimulating tissue healing for burns or soft tissue defects while also reducing contamination. The results of this study will advise future generations of this product, specifically regarding selecting C2DA and BUP loading concentrations, allowing preclinical and clinical studies to further characterize efficacy.

## Figures and Tables

**Table 1:** Loading concentrations and abbreviations for each DA-ESCM group.

	C2DA Loading	BUP Loading
Unloaded	-	-
Low C2DA	0.075 mg	-
Med C2DA	0.15 mg	-
High C2DA	0.3 mg	-
Low BUP	-	0.25 mg
Med BUP	-	0.5 mg
High BUP	-	1.0 mg
Low combo	0.075 mg	0.25 mg
Medium combo	0.15 mg	0.5 mg
High combo	0.3 mg	1.0 mg

## Data Availability

Data available on request.
